# High salt induces P-glycoprotein mediated treatment resistance in breast cancer cells through store operated calcium influx

**DOI:** 10.18632/oncotarget.25391

**Published:** 2018-05-18

**Authors:** Duaa Babaer, Suneetha Amara, Michael Ivy, Yan Zhao, Philip E. Lammers, Jens M. Titze, Venkataswarup Tiriveedhi

**Affiliations:** ^1^ Department of Biological Sciences, Tennessee State University, Nashville, TN, USA; ^2^ Department of Medicine, St Thomas-Midtown Hospital, Nashville, TN, USA; ^3^ Division of Clinical Pharmacology, Vanderbilt University Medical Center, Nashville, TN, USA; ^4^ Department of Medicine, Meharry Medical College, Nashville, TN, USA; ^5^ Cardiovascular and Metabolic Disorders program, Duke-NUS Medical School, Singapore; ^6^ Department of Pharmacology, Vanderbilt University, Nashville, TN, USA

**Keywords:** breast cancer, salt, P-glycoprotein, store operated calcium entry, prostratin

## Abstract

Recent evidence from our laboratory has demonstrated that high salt (Δ0.05 M NaCl) induced inflammatory response and cancer cell proliferation through salt inducible kinase-3 (SIK3) upregulation. As calcium influx is known to effect inflammatory response and drug resistance, we examined the impact of high salt on calcium influx in breast cancer cells. Treatment of MCF-7 and MDA-MB-231 cells with high salt induced an enhanced intracellular calcium intensity, which was significantly decreased by store operated calcium entry (SOCE) inhibitor co-treatment. Further, high salt induced P-glycoprotein (P-gp) mediated paclitaxel drug resistance in breast cancer cells. Murine tumor studies demonstrated that injection of MCF-7 cells cultured in high salt, exerted higher tumorigenicity compared to the basal cultured counterpart. Knock down of SIK3 by specific shRNA inhibited tumorigenicty, expression of SOCE regulators and P-gp activity, suggesting SIK3 is an upstream mediator of SOCE induced calcium influx. Furthermore, small molecule inhibitor, prostratin, exerted anti-tumor effect in murine models through SIK3 inhibition. Taken together, we conclude that SIK3 is an upstream regulator of store operated calcium entry proteins, Orai1 and STIM1, and mediates high salt induced inflammatory cytokine responses and P-gp mediated drug resistance. Therefore, small molecule inhibitors, such as prostratin, could offer novel anti-cancer approaches.

## INTRODUCTION

Breast cancer is the second leading cause of cancer related mortality in American women [1]. Despite the significant improvement in both diagnostic and therapeutic modalities for the treatment of cancer patients, about 30% of patients with early-stage breast cancer succumb to recurrent disease [2]. While, systemic anti-cancer agents are effective at the beginning of therapy both in primary breast cancers and metastases, however, after a variable period of time, resistance to therapy is common mainly due to emergence of tumor variance phenotypes [3]. Although the elimination of transformed cells by host immune responses result in immune sculpting, and emergence of new treatment resistant tumor variants, the exact mechanisms and molecular factors in the tumor microenvironment mediating this cancer resistance are yet undefined [4].

Recent evidence from our laboratory has demonstrated that under high salt (50 mM above basal condition Δ0.05 M NaCl) external treatment conditions, breast cancer cells induce chronic inflammatory response with enhanced expression of inflammatory cytokines and reactive oxygen/nitrogen species (RNS/ROS) [5, 6]. Interestingly, sodium-magnetic resonance imaging (Na^23^-MRI) studies performed in breast cancer patients have demonstrated an increased sodium content of up to 50–70% in breast tumors as compared with surrounding soft tissue [7, 8]. These Na^23^-MRI studies in corroboration with our *in vitro* data argue for a potential effector role of salt in the tumor microenvironment towards promotion of tumor progression and probably treatment resistance in breast cancer cells.

Calcium influx mediated signaling response is well known to induce expression and secretion of inflammatory cytokines [9]. Altered expression of STIM1 and Orai1, key molecular components of store operated calcium entry (SOCE) pathways have been reported in cervical cancer [10], breast cancer [11], and esophageal cancer [12]. Further, P-glycoprotein upregulation is well known to induce treatment resistance in cancer cells. P-glycoprotein is a product of the multi drug resistance gene complex (MDR) and functions as an energy-dependent drug efflux pump and acts by active intra-cellular removal of anti-cancer drugs and there by development of treatment-resistant tumor variants [13]. In our current communication, we studied the potential role of high salt treatment towards induction of calcium influx mediated inflammatory signaling and its interplay towards induction of P-glycoprotein mediated treatment resistance.

## RESULTS

### Store operated calcium channels are critical for high salt mediated inflammatory cytokine release

**Figure 1:**
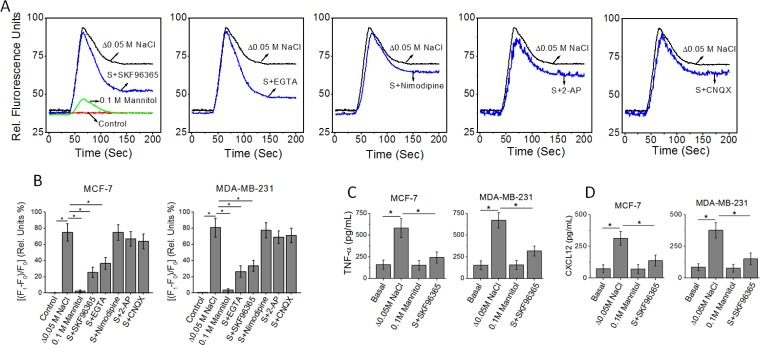
(**A**) Fluo-3 Ca^2+^ measurement indicates that SKF96365 (10 μM, inhibitor of store operated Ca^2+^ entry) and EGTA (2 mM) treatments decrease high salt (Δ0.05 M NaCl) induced Ca^2+^ influx in MCF-7 breast cancer cells. The basal (normal salt) treatment is indicated in red as control. While inhibitors of voltage-gated Ca^2+^ channels (nimodipine, 10 μM), NMDA receptors (2-AP, 10 μM), or AMPA receptors (CNQX, 10 μM) had no effect on high salt-induced calcium influx. (**B**) Quantitative changes high salt induced calcium influx measured by relative florescence shift (ΔF/F*100) in MCF-7 and MDA-MB-231 breast cancer cells following various treatment conditions. F1, plateau phase fluorescence; F0, baseline fluorescence. (**C**, **D**) Inhibition of inflammatory cytokine TNF-α (C), and inflammatory chemokine CXCL12 (D) expression MCF-7 and MDA-MB-231 breast cancer cells in following treatment with SOCE inhibitor. Data were representative of five experiments and shown as mean ± SEM, *p <* 0.05.

We have previously demonstrated that high salt treatment (Δ0.05 M NaCl) induced expression of inflammatory cytokines by breast cancer cells [5]. As the ubiquitous second messenger, Ca^2+^, is one of the critical regulators of inflammatory responses, we investigated the interplay of Ca^2+^ influx on high salt mediated cytokine release [14]. Towards this we first performed a Fluo-3 (a fluorescent Ca^2+^ indicator)-based Ca^2+^ measurement, to determine the induction of calcium influx following high salt treatment on breast cancer cell lines, MCF-7 and MDA-MB-231. As shown in Figure 1, high salt treatment induced an enhanced calcium influx peak. Normally, the cytoplasmic calcium influx peak consists of two phases, a peak phase contributed by Ca^2+^ release from intracellular Ca^2+^ stores and a plateau phase contributed by Ca^2+^ influx. As shown in Figure 1A, SKF96365, an inhibitor of store operated Ca^2+^ entry (SOCE) [15], decreased the amplitude of the plateau phase of the high salt-induced Ca^2+^ response without affecting the peak phase. Similar results were observed with EGTA, which chelates extracellular Ca^2+^. Quantitative analysis of the fluorescence intensity changes of the plateau phase demonstrated that high salt induced a 76 ± 10% calcium influx induced Fluo-3 intensity change. Here, 0.1 M mannitol is used as a negative control for the high salt (0.05 M NaCl) treatment. NaCl being a bi-ionic species the ionic osmolar equivalent of 0.05 M NaCl is 0.1 M mannitol. As shown in Figure 1A, treatment of cancer cells with equivalent mannitol concentration did not induce a calcium response, and thus suggesting that the calcium signal changes were a direct consequence of salt induced phenomenon and not a secondary effect as consequence of osmolar-changes induced by high salt. Interestingly, SKF96365 decreased the change (24 ± 6%, *p <* 0.05) in plateau phase of calcium influx, similar to the effect shown by EGTA. However, inhibitors of voltage-gated Ca^2+^ channels (nimodipine), NMDA receptors (2-AP), or AMPA receptors (CNQX) had minimal effect (Figure 1A and 1B). Further, ELISA based analysis of the TNF-α (Figure 1C) in the cell supernatant from MCF-7 cells following high salt treatment was determined to be 583 ± 109 pg/mL (*p <* 0.05, compared to basal normal salt treatment which is 161 ± 109 pg/mL). However, with SKF96365, a SOCE specific inhibitor treatment under high salt conditions induced inhibition of TNF-α secretion (243 ± 64 pg/mL, *P <* 0.05 compared to high salt treatment). Similarly, ELISA based analysis of the CXCL12 (Figure 1D) in the cell supernatant from MCF-7 cells following high salt treatment was determined to be 314 ± 54 pg/mL (*p <* 0.05, compared to basal normal salt treatment which is 73 ± 32 pg/mL). However, with SKF96365, a SOCE specific inhibitor treatment under high salt conditions induced inhibition of CXCL12 secretion (138 ± 44 pg/mL, *P <* 0.05 compared to high salt treatment). Collectively, the inhibition of SOCE demonstrated decreased expression of high salt-induced release of inflammatory cytokine and chemokine, TNF-α and CXCL12, respectively, in the breast cancer cells MCF-7 and MDA-MB-231 (Figure 1C, 1D). Therefore, these data suggest that high salt induces its inflammatory response through upregulation of SOCE mediated Ca^2+^ influx.

### STIM1 and Orai1 are required for high salt mediated inflammatory response

**Figure 2:**
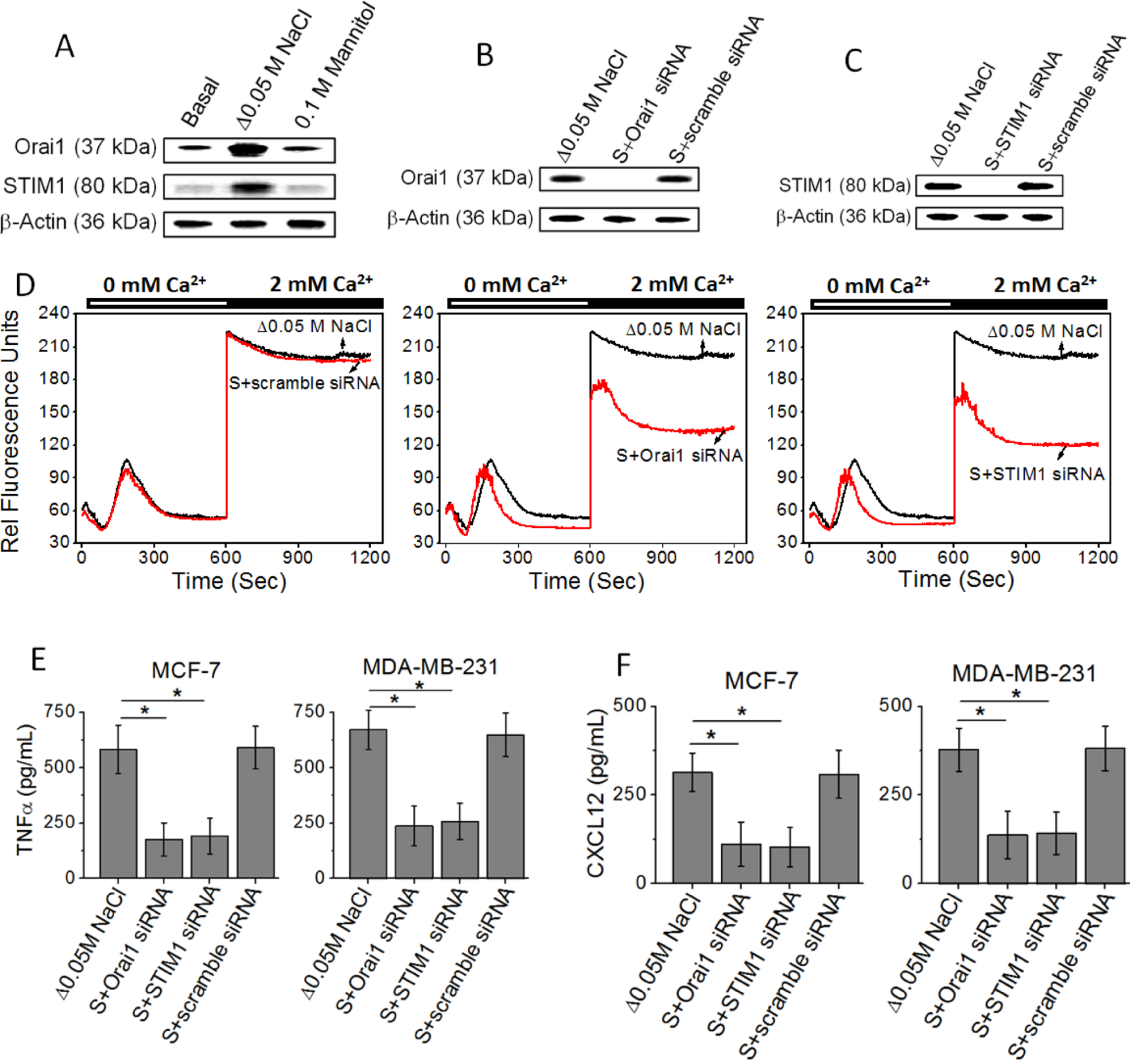
**(A)** Western blot analysis to demonstrate the expression of Orai1 and STIM1 followinh high salt treatment (Δ0.05 M NaCl). **(B, C)** Inhibition of Orai1 (B) and STIM1 (C) following high salt+specific siRNA treatment. **(D)** Fluo-3 Ca^2+^ measurement indicates that STIM1 siRNA and Orai1 siRNA decrease store-operated Ca^2+^ influx in MCF-7 cells. **(E, F)** Inhibition of inflammatory cytokine TNF-α (E), and inflammatory chemokine CXCL12 (F) expression MCF-7 and MDA-MB-231 breast cancer cells in following siRNA based knock down of Orai1 and STIM1. Data were representative of five experiments and shown as mean ± SEM, *p <* 0.05.

Several studies from other laboratories have demonstrated that the proteins, STIM1 and Orai1, are responsible for store-operated Ca^2+^ entry [16]. To examine whether STIM1 and Orai1 are important molecular components involved in high salt mediated inflammatory responses, we used RNA interference (RNAi) to knock down STIM1 and Orai1 in MCF-7 and MDA-MB-231 human breast cancer cells. The successful knockdown of STIM1 or Orai1 mRNA was confirmed by western blot (Figure 2A–2C). Furthermore, the reduction of functional store-operated Ca^2+^ entry was verified by Fluo-3-based measurements (Figure 2D). Store-operated Ca^2+^ channels are activated when internal Ca^2+^ stores are empty. Thapsigargin was used to empty the intracellular Ca^2+^ stores in the absence of extracellular Ca^2+^. The Ca^2+^ influx was then measured by addition of 2 mM extracellular Ca^2+^. Both STIM1 and Orai1 siRNAs reduced the level of Ca^2+^ influx compared to control scramble siRNA (Figure 2D). Further, importantly, knock down of STIM1 and Orai1 inhibited the high salt mediated inflammatory response (Figure 2E and 2F) by MCF-7 and MDA-MB-231. Therefore, these data demonstrate that SOCE mediated Ca^2+^ influx is critical for the high salt induces its inflammatory response in breast cancer cells.

### High salt induces upregulation of P-glycoprotein, a drug resistance efflux pump

**Figure 3:**
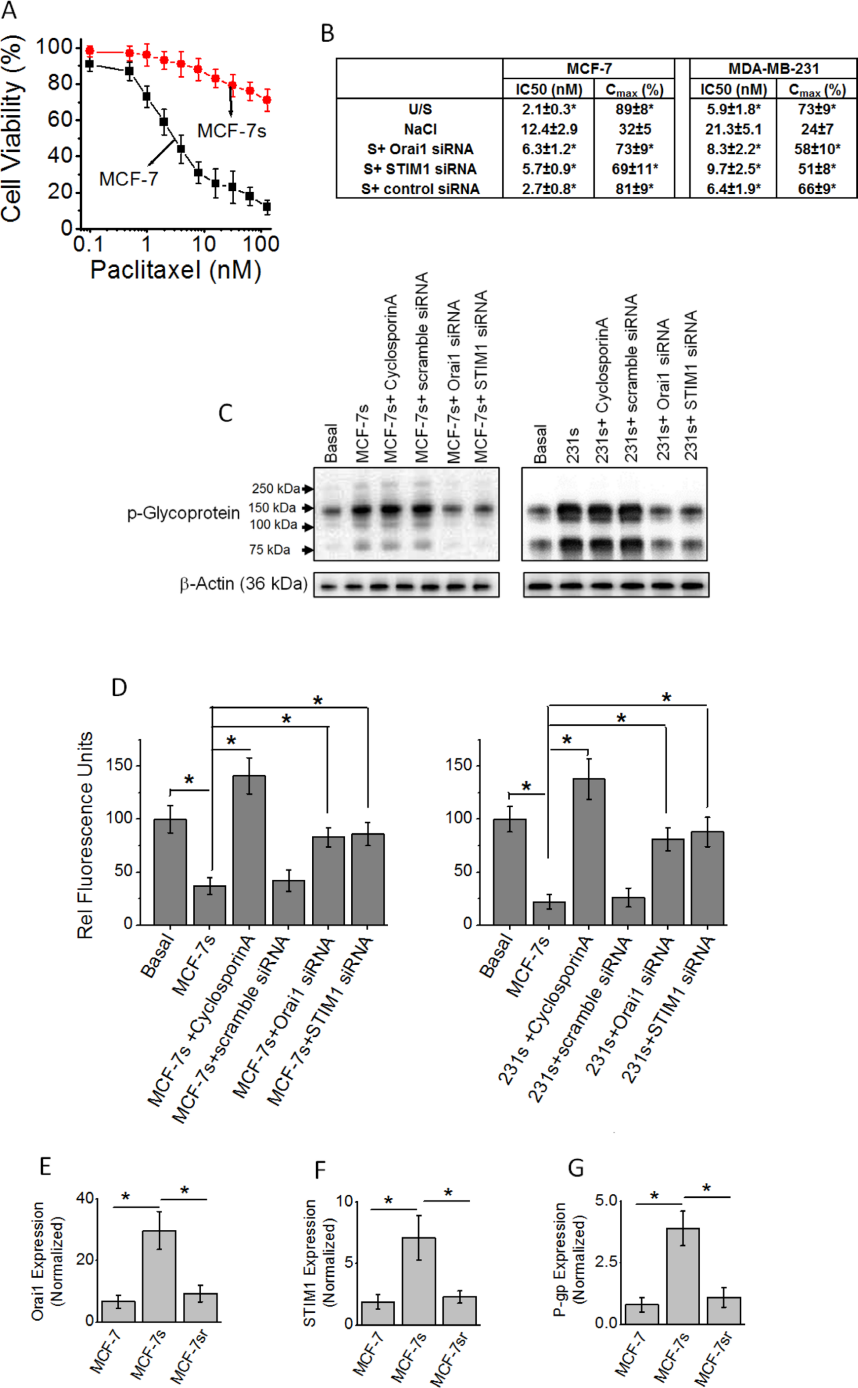
(**A**) Impact of high salt treatment on paclitaxel resistance. MCF-7 cells were cultured under basal conditions; MCF-7s cells were passaged eight times in high salt added cell culture media (Δ0.05 M NaCl). (**B**) Pharmacokinetic parameters of paclitaxel cytotoxicity following various treatment conditions. (**C**) Western blot analysis of P-glycoprotein expression following high salt treatment. MCF-7s, represent MCF-7 cells were passaged eight times in high salt added cell culture media (Δ0.05 M NaCl); 231s, represents MDA-MB-231 cells passaged eight times in high salt added cell culture media (Δ0.05 M NaCl). (**D**) Impact of high salt treatment on intracellular Rhodamine-123 accumulation. Rhodamine 123 is pumped out of the cell during enhanced expression of P-glycoprotein. (**E–G**) The high salt treated cells (for 8 passages) were latter cultured in normal salt containing basal media for 5 passages (referred to as MCF-sr) and then tested for the expression of Orai1 (E), STIM1 (F) and P-glycoprotein (G). Data were representative of five experiments and shown as mean ± SEM, *p <* 0.05.

Our previous studies have demonstrated that high salt treatment (Δ0.05 M NaCl) along with inflammatory stress (as noted by enhanced reactive nitrogen and oxygen species) has also induced a 24% proliferation of cancer cells compared to basal culture conditions [5]. As chronic inflammatory stress is known to result in cancer cell variants, we next performed experiments to determine if high salt also induces treatment resistance phenotype in breast cancer cells [17]. Towards this, we have passaged MCF-7 and MDA-MB-231 cells in high salt for eight passages and will be referred to as MCF-7s and 231s, respectively. As shown in Figure 3A and 3B, high salt treated MCF-7s were resistant to paclitaxel induced cytotoxicity as measured by IC50 and C_max_ compared to basal culture conditions. Further literature evidence suggests that the emergence of chemotherapeutic multidrug resistance (MDR) was associated with increased levels of a transmembrane glycoprotein, P-glycoprotein (P-gp). Therefore, we tested if there is change in P-gp expression following high salt treatment. As shown in Figure 3C, high salt treatment in both MCF-7s and 231s cell lines, induced enhanced expression of chemoresistance factor, P-gp. Further, it is important to note that knock down of SOCE Ca^2+^ regulator molecules STIM1 and Orai1-enhanced paclitaxel sensitivity (Figure 3B) and reduced the expression of expression of P-gp (Figure 3C). This suggests that high salt mediated calcium influx regulates the development of drug resistance in salt passaged breast cancer cells. Further, to determine the functionality of the high salt mediated expression of P-gp, a membrane drug efflux channel, we performed rhodamine 123 efflux assay. The cells are pre-treated with rhodamine 123 for 1 hour, and then washed and measured for intracellular accumulation of the dye. The P-gp is a specific membrane protein which facilitates the efflux of rhodamine123, and cells with higher expression of P-gp have poor intracellular accumulation of rhodamine 123. As shown in Figure 3D, high salt passaged cells (MCF-7s and 231s) demonstrated decreased cellular accumulation of rhodamine 123 (37 ± 8 RFU and 22 ± 7 RFU, respectively) compared to breast cancer cells cultured under basal condition (rhodamine efflux in MCF-7 and MDA-231 is normalized 100 RFU). In the presence of cyclospoin-A, a known specific P-gp inhibitor [18], and P-gp knock down there is enhanced intracellular rhodamine 123 accumulation in the high salt passaged cancer cells, thus clearly suggesting that the drug efflux functionality of P-gp is specifically upregulated in the high salt mediated drug resistance. Importantly, knock down of SOCE calcium regulators STIM1 and Orai1 (Figure 3D) increased intracellular rhodamine 123 accumulation and thus arguing for a critical role of SOCE mechanism in high salt mediated P-gp expression leading to drug resistance. To test for the reversible nature of the SOCE expression under normal salt (basal condition) treatment following high salt treatment (for 8 passages), we have cultured the MCF-7s cells in regular basal conditions for 5 passages (these cells will be referred as MCF-7sr cells). As shown in Figure 3E–3G, reversing the salt treatment conditions to basal levels in MCF-7sr cells reversed the expression of Orai1, STIM1 and P-glycoprotein to basal levels. These data suggest that the high induced SOCE expression and P-glycoprotein mediated drug resistance is a reversible phenomenon.

### Enhanced tumorigenicity of high salt pre-treated breast cancer cells

**Figure 4:**
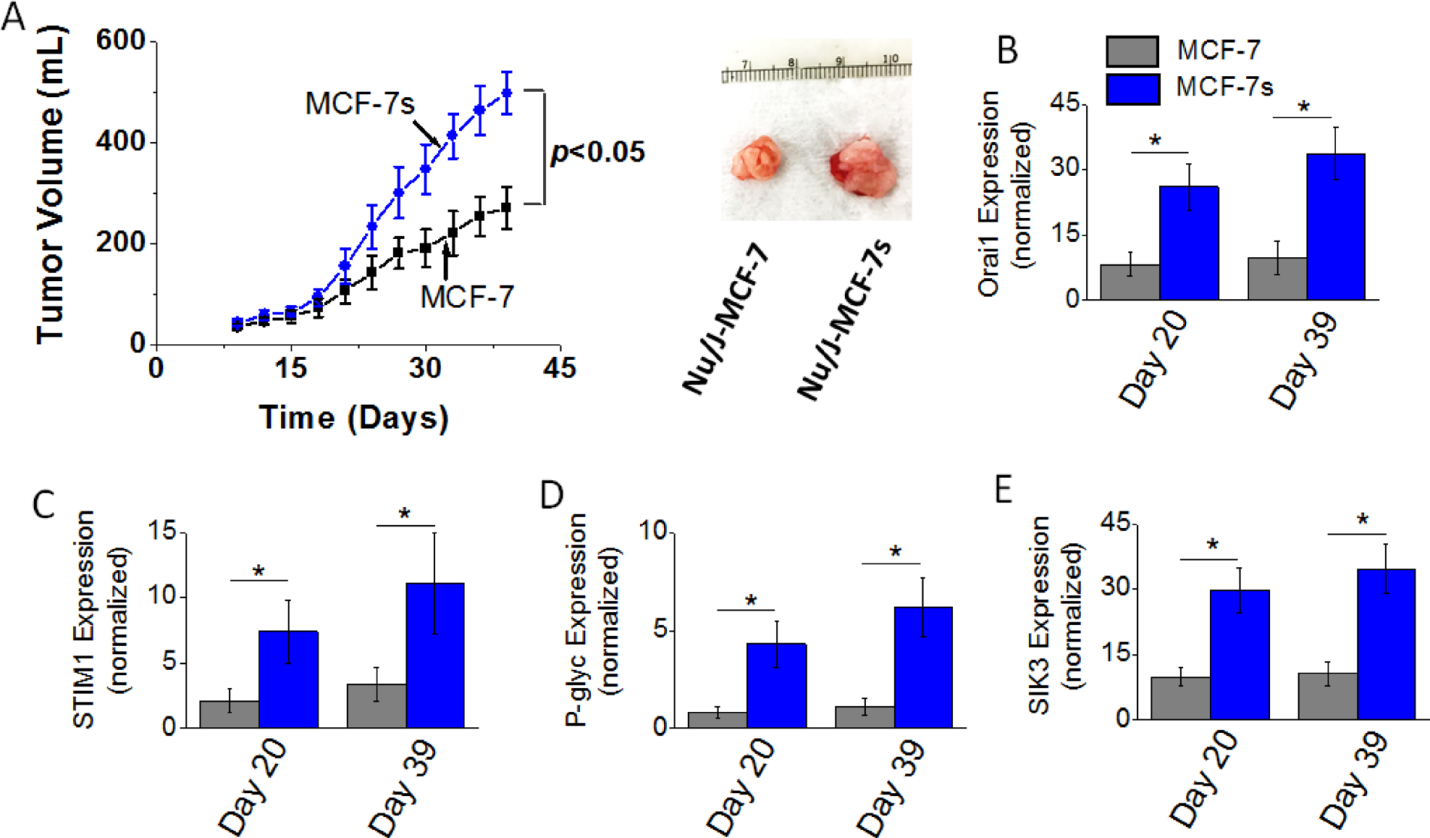
Tumorigenicity of MCF-7 and MCF-7s breast cancer cells (**A**) Temporal changes in the tumor volume following injection of 5 × 10^5^ MCF-7 and MCF-7s cells into Nu/J (*n* = 6) mice. (**B**–**E**) The mRNA expression of Orai (B), STIM1 (C), P-glycoprotein (D), and SIK3 (E). Data were represented as mean ± SEM, *n* = 6 per cohort, *p <* 0.05.

To determine the effect of high salt passage on tumorigenicity of breast cancer cells we performed *in vivo* studies. Orthotopic tumors were induced following intramammary injection of 5 × 10^5^ cancer cells (MCF-7 or MCF-7s) in Nu/J mice. As shown in Figure 4A, tumor volume at the end of day 39 in mice injected with high salt passaged MCF-7s cells was 498 ± 41 mm^3^; while tumor volume in mice injected by MCF-7 cells on day 39 was 271 ± 43 mm^3^ (*p <* 0.05). Further, mRNA based gene expression analyses (Figure 4B–4D) demonstrated an enhanced expression of SOCE regulatory molecules Orai1 and STIM1, and drug resistance protein P-gp. Using cell based phosphoproteomics approach and further functional analyses; we have previously demonstrated salt inducible kinase-3 (SIK3) mediates high salt mediated cell proliferation and inflammatory responses. In our current *in vivo* studies, we further confirm that in the orthotopic MCF-7s tumors there is 3.3 (*p* < 0.05) fold enhanced expression of SIK3 (Figure 4E) compared to orthotopic MCF-7 induced tumors. These data suggest that high salt pretreatment enhances the cell proliferation and tumorigenicity of breast cancer cells.

### Decreased tumorigenicity following knock down of Orai1 in high salt pre-treated breast cancer cells

**Figure 5:**
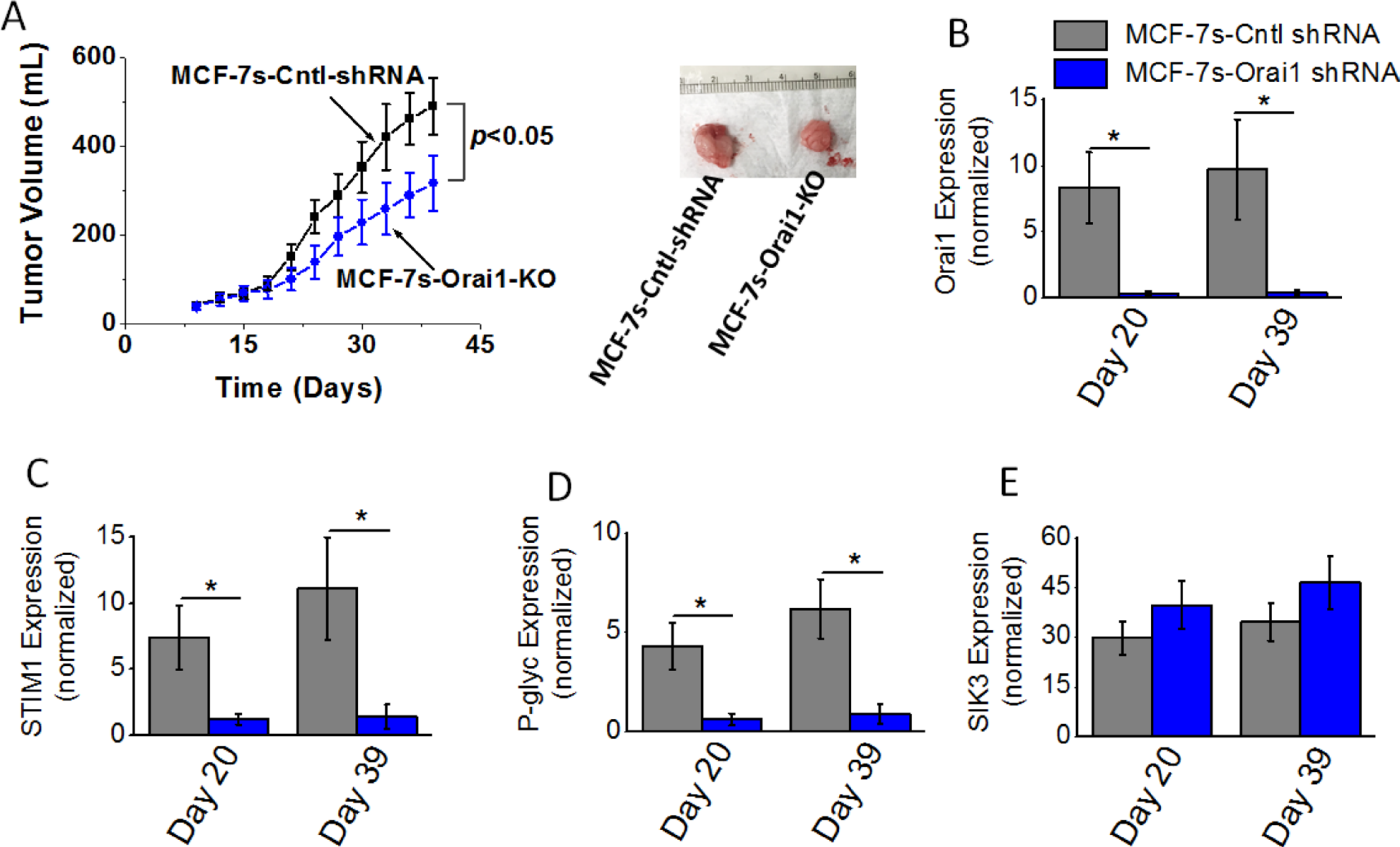
Tumorigenicity of high salt passaged breast cancer cells following shRNA knock down of Orai1 expression (**A**) Temporal changes in the tumor volume following injection of 5 × 10^5^ MCF-7 and MCF-7s cells into Nu/J (*n* = 6) mice. (**B**–**E**) The mRNA expression of Orai (B), STIM1 (C), P-glycoprotein (D) and SIK3 (E). Data were represented as mean ± SEM, *n* = 6 per cohort, *p <* 0.05.

As SOCE mediated calcium influx regulator Orai1 played a critical role in high salt mediated inflammatory response and paclitaxel resistance, we next tested the impact of Orai1 knock down on the *in vivo* tumorigenicity of breast cancer cells. Orthotopic tumors were induced with MCF-7s control shRNA treated (MCF-7s-Cntl-shRNA) or Orai1 shRNA treated (MCF-7s-Orai1-KO) in Nu/J mice. As shown in Figure 5A, tumor volume at the end of day 39 in mice injected with high salt passaged MCF-7s cells was 483 ± 64 mm^3^; while tumor volume in mice injected by Orai1-KO-MCF-7s cells on day 39 was 316 ± 72 mm^3^ (*p <* 0.05). Further, mRNA based gene expression analyses (Figure 5B–5D) demonstrated a diminished expression of SOCE regulatory molecules Orai1 and STIM1, and drug resistance protein P-gp. However, SIK3 expression (Figure 5E) remained unchanged with or without Orai1 knock down in MCF-7s cell induced tumors. These data indicated to us that SIK3 is upstream to SOCE regulators in high salt mediated tumorigenicity of breast cancer cells.

### Critical role of SIK3 in high salt mediated enhanced tumorigenicity

**Figure 6:**
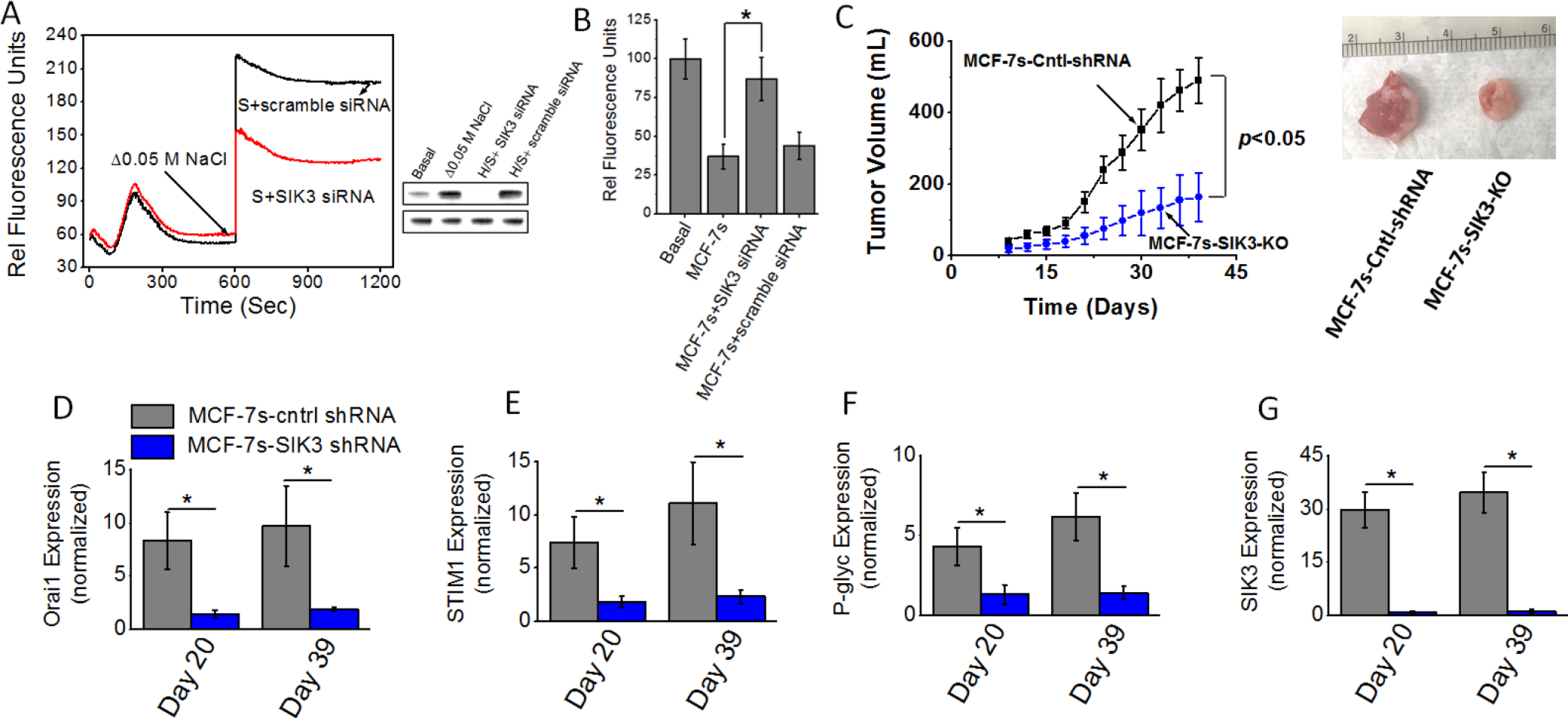
(**A**) Fluo-3 Ca^2+^ measurement following siRNA knock down of SIK3 in MCF-7 cells. Westernblot analysis to demonstrate SIK3-siRNA knock down efficiency (H/s refers to high salt). (**B**) Impact of high salt treatment plus SIK3 knock down on intracellular Rhodamine-123 accumulation. (**C**) Tumorigenicity of high salt passaged breast cancer cells following shRNA knock down of SIK3 expression. Temporal changes in the tumor volume following injection of 5 × 10^5^ MCF-7 and MCF-7s cells into Nu/J (*n* = 6) mice. (**D–G**) The mRNA expression of Orai (D), STIM1 (E), P-glycoprotein (F) and SIK3 (G). Data were represented as mean ± SEM, *n* = 6 per cohort, *p <* 0.05.

Previous phosphoproteomics based data from our laboratory demonstrated an enhanced expression and phosphorylation of SIK3 in breast cancer cells following high salt treatment. Further, we have shown that SIK3 mediates G0/G1-cell cycle release leading to mitotic cell division and cell proliferation [6]. Our initial studies (Figures 4 and 5) have demonstrated an enhanced expression of SIK3 in high salt passaged MCF-7s breast cancer cells. Therefore, we tested if SIK3 plays a role in SOCE mediated calcium influx following high salt treatment. As shown in Figure 6A, 6B, thapsigargin emptied intracellular Ca^2+^ stores in the absence of extracellular Ca^2+^, followed by the Ca^2+^ influx measured by addition of 2 mM extracellular Ca^2+^ demonstrated that knock down of SIK3 reduced calcium influx and thus suggesting that SIK3 plays a direct role in calcium influx. To determine the tumorigenicity, we injected shRNA mediated SIK3 knock out MCF-7s cells into Nu/J mice. As shown in Figure 6C, tumor volume at the end of day 39 in mice injected with high salt passaged MCF-7s cells was 478 ± 79 mm^3^; while tumor volume in mice injected by SIK3-KO-MCF-7s cells on day 39 was 164 ± 57 mm^3^ (*p <* 0.05). Further, mRNA based gene expression analyses (Figure 6D–6G) demonstrated a diminished expression of SIK3, SOCE regulatory molecules Orai1 and STIM1, and drug resistance protein P-gp. These data clearly demonstrated to us that SIK3 is upstream signaling molecule to SOCE regulated calcium influx in high salt mediated tumorigenicity of breast cancer cells.

### Specific anti-tumor effect of prostratin on high salt treated breast cancer cells

**Figure 7:**
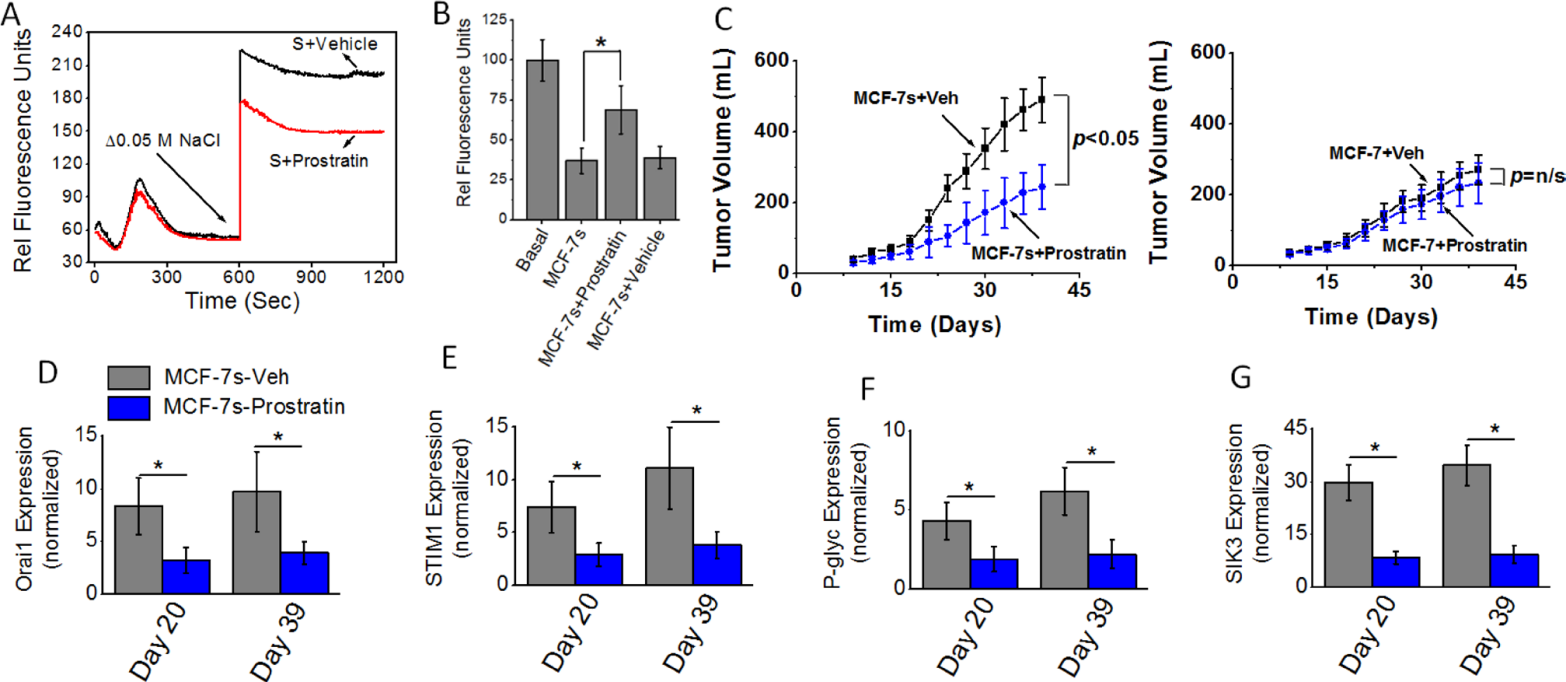
(**A**) Fluo-3 Ca^2+^ measurement following Prostratin (8 μM) plus high salt treatment on MCF-7 cells. (**B**) Impact of high salt plus Prostratin (8 μM) treatment on intracellular Rhodamine-123 accumulation. (**C**) Tumorigenicity of high salt passaged breast cancer cells following oral administration of prostratin (100 μM). Temporal changes in the tumor volume following injection of 5 × 10^5^ MCF-7 and MCF-7s cells into Nu/J (*n* = 6) mice. (**D–G**) The mRNA expression of Orai (D), STIM1 (E), P-glycoprotein (F) and SIK3 (G). Data were represented as mean ± SEM, *n* = 6 per cohort, *p <* 0.05.

We have recently demonstrated that prostratin, a small molecule identified in the tea from the bark of Somoan tree and extensively studied in HIV research, induced a cytotoxic effect specifically on high salt treated breast cancer cells potentially through SIK3 inhibition [19]. We tested if prostratin could inhibit calcium influx and the tumorigenicity of high salt passaged breast cancer cells. We have orally administered prostratin (100 μM) into Nu/J mice two weeks prior to injection of MCF-7s cells. As shown in Figure 7A and 7B, thapsigargin emptied intracellular Ca^2+^ stores in the absence of extracellular Ca^2+^, followed by the Ca^2+^ influx measured by addition of 2 mM extracellular Ca^2+^ along with 8 μM prostratin demonstrated reduced calcium influx compared with vehicle control treated MCF-7 cells. Further, we orally administered prostratin (100 μM) into Nu/J mice two weeks prior to injection of MCF-7s cells. As shown in Figure 7C, tumor volume at the end of day 39 in mice injected with vehicle treated high salt passaged MCF-7s cells was 491 ± 68 mm^3^; while tumor volume in mice injected by prostratin treated MCF-7s cells on day 39 was 245 ± 62 mm^3^ (*p <* 0.05). However, prostratin treatment in MCF-7 cells cultured under basal conditions did not demonstrate inhibition of tumorigenicity. Further, mRNA based gene expression analyses (Figure 7D–7G) demonstrated a diminished expression of SIK3, SOCE regulatory molecules Orai1 and STIM1, and drug resistance protein P-gp. These data clearly demonstrated to us that prostratin exerts anti-tumor effect specifically on high salt pre-treated breast cancer cells possibly through inhibition of SIK3-SOCE signaling.

## DISCUSSION

Ca^2+^ is one of the most important signaling molecules known to regulate multiple cellular processes including, transcription, secretion of intracellular cytokines and cell division [20]. Multiple lines of evidence strongly suggests that cancer cells upregulate intracellular Ca^2+^-related signaling along with constant alterations in the expression and/or activity of calcium channels and pumps on the cell membrane [21]. Cancer cells are considered to undergo these changes to sustain their own cellular proliferation and to avoid cell death events. Human sodium-MRI evidence demonstrated high sodium concentration in breast tumors [7], although the functional significance of this high sodium in tumor microenvironment is unknown. While the exact reasons for the high sodium concentration in tumor microenvironment are unknown, previous studies from our laboratory demonstrated that high salt treatment on breast cancer cells induced inflammatory and cell proliferative responses [5, 22, 23]. As calcium signaling is one of the key mediators of these cellular responses too, in this study we evaluated if high salt treatment induces Ca^2+^ influx. Our current studies demonstrated that high salt treatment in breast cancer cells induced inflammatory response through SOCE regulated Ca^2+^ influx mediated signaling. Our current cancer cellular evidence corroborates well with evidence by Yang *et al*., wherein, they have shown SOCE mediated signaling is critical for the invasiveness of breast cancer cells [24]. These data suggest that SOCE regulated Ca^2+^ influx mediated signaling plays a critical role in multiple pro-cancer cellular processes. Further studies are needed to confirm our observations utilizing complete and conditional STIM1 and Orai1 knock out murine models. Also in it important to note that STIM1 expression is influenced by temperature changes [25]. As inflammatory responses could induced local changes in the basal cellular temperatures, future studies are needed to study the impact of temperature on high salt induced SOCE expression.

**Figure 8:**
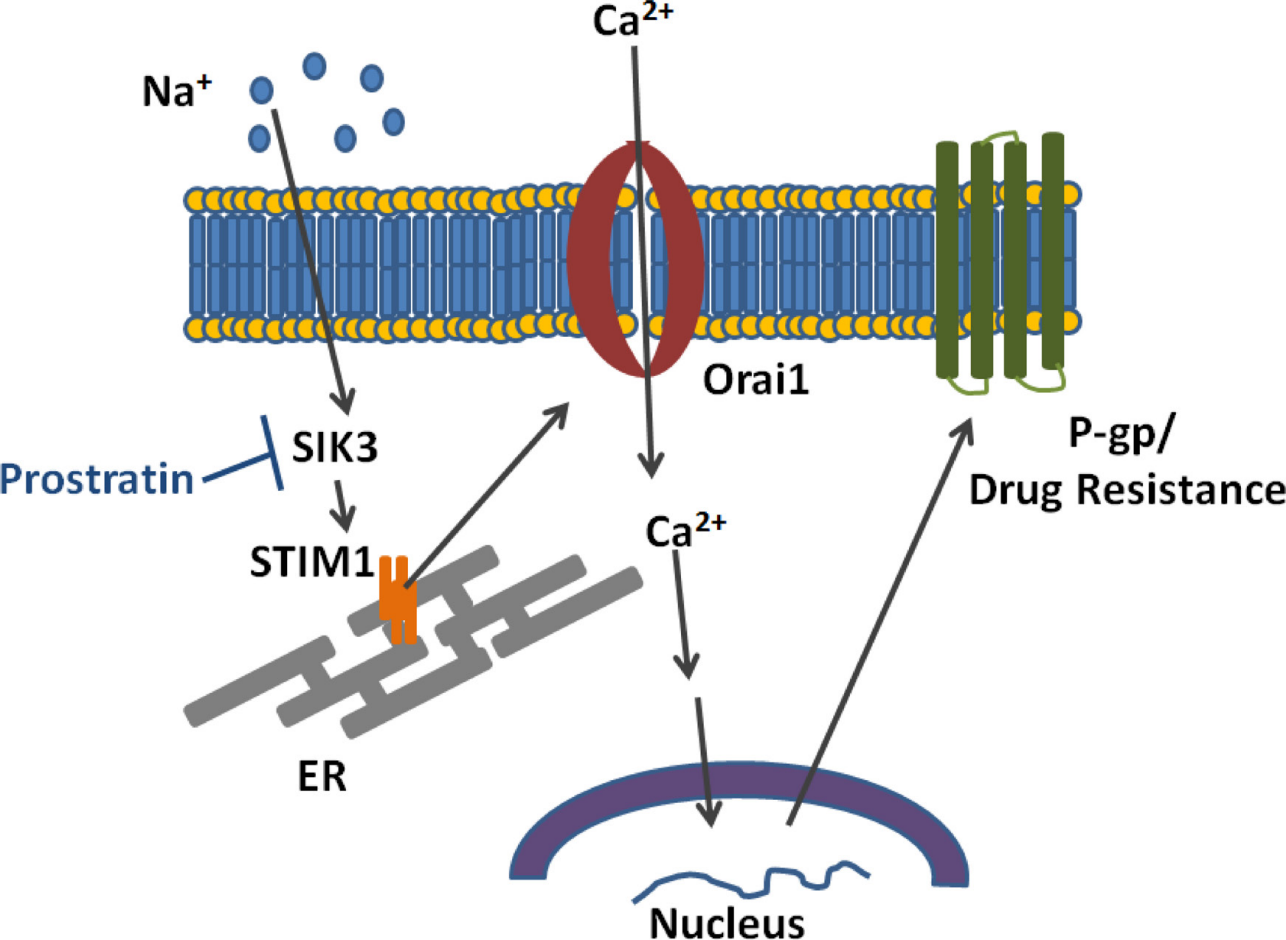
Schematic on the mechanism by which high salt induces calcium influx and P-glycoprotein mediated drug resistance

The overexpression of P-gp is one of the well-known mechanisms by which breast cancers cells develop chemo-drug resistance [26]. In this study we investigated if high salt treatment induces expression of P-gp. We demonstrated that high salt induces paclitaxel resistance through SOCE regulated Ca^2+^ influx mediated signaling P-gp overexpression (Figure 8). Xie *et al.* have demonstrated that the expression of P-gp is also regulated by post-translational events, such as post-transcriptional glycosylation and membrane localization of P-gp [27]. Therefore, using rhodamine 123 assay we demonstrated that high salt induces stable localization of P-gp and thus possibly inhibiting paclitaxel mediated cytotoxicity on breast cancer cells. Complex functional mechanisms have been noted between the P-gp-mediated drug resistance and intracellular calcium homeostasis. Our current studies support the previous studies by Gibalova *et al*., where in they demonstrated that the Ca^2+^ influx was enhanced in cells with P-gp overexpression [28]. Further, a higher intracellular calcium concentration was noted in P-gp-positive MCF-7 breast cancer cells as compared with its P-gp-negative MCF-7 cells [29]. Taken together, our current studies support a notion that high salt in the tumor microenvironment induce P-gp mediated drug resistance in breast cancer.

SOCE regulate Ca^2+^ signaling has been implicated in various oncological processes including dysplasia, metastasis and angiogenesis [30]. SOCE is also known to induce extracellular secretion of vascular endothelial growth factor (VEGF), which increase tumor vascularity and tumor progression [31]. While SOCE in cancer cells is known to promote tumorigenicity, SOCE mediated Ca^2+^ signaling in known to activate cytotoxic CD8+T-lyphocytes which induce anti-tumor effect [32]. Therefore, to specifically study the tumorigenic role of high salt induced SOCE in cancer cells we performed *in vivo* studies in the immunodeficient Nu/J mice. Our *in vivo* studies demonstrated that high salt pre-treated breast cancer cells display enhanced tumorigenicity compared with breast cancer cells passaged under basal media culture conditions. These data point out an important functional role of high salt towards breast tumor progression.

Previous phosphoproteomics based studies from our laboratory demonstrated overexpression and phosphorylation of SIK3 following high salt treatment [6]. Further we reported that prostratin, a small molecule inhibitor, known to inhibit HIV reactivation in CD4+T-lymphocytes, plays an important role in SIK3inhibition [19]. In our current study, we demonstrated that prostratin was specifically effective against high salt pre-treated breast cancer cells. This supports our previous *in vitro* studies wherein we demonstrated that prostratin was upto 5 fold more cytotoxic on high salt pre-treated breast cancer cells.

In conclusion, we determined the molecular functionality of high salt on breast cancer cells towards induction of their tumorigenicity and drug resistance through SOCE regulated Ca^2+^ influx. The Orai1 and STIM1 inhibition could offer novel anti-cancer therapeutic strategies. Further, our current evidence to support the anti-tumor effect of prostratin could evoke further research to study the potential novel application of this small molecule inhibitor as an independent or add-on drug to the current breast cancer treatment regimen.

## MATERIALS AND METHODS

### Cell culture

Breast cancer cells (MCF7 and MDA-MB-231) were utilized and obtained from the American Type Culture Collection (ATCC, Manassas, VA) and cultured in cell basal essential media (RPMI1640 media, Thermo Fisher Scientific, Waltham, MA) along with the media supplements such as fetal bovine serum, penicillin/streptomycin, fungizone, HEPES and glutamine, as recommended by the manufacturer and as previously described [5, 33]. Cell lines were frozen in liquid vapor nitrogen at –130° C until use. Upon thawing, cells were maintained in 5% CO_2_ incubator in sterile essential media at 37° C. For salt treatment conditions, cell culture media was supplemented with 0.05 M NaCl (Sigma Aldrich, St Louis, MO). We have previously performed a dose-response for salt (0–0.1 M NaCl) and found-out that 0.05 M NaCl provided highest cell proliferation [5, 23]. All chemicals unless mentioned were obtained either from Sigma Aldrich (St Louis, MO) or Thermo Fisher Scientific (Waltham, MA). The siRNA constructs against human STIM1 and Orai1 were generated using the pSUPER.retro vector according to the manufacturer's instructions (OligoEngine). The sequences used were 5′-GGCTCTGGATACAGTGCTC-3′ for STIM1 and 5′-CGTGCACAATCTCAACTCG-3′ for Orai1; 5′-GTGCAGAGTGTTGGAGTCC -3′ for SIK3. siRNA-transfected cells were selected using puromycin. For murine injections cells were transfected by the following shRNA: Orai shRNA (sc-76001-SH, Santa Cruz Biotech), and SIK3 shRNA (sc-97056-SH, Santa Cruz Biotech). Prostratin (Sigma Aldrich) treatment on cell lines was performed to determine the effect of the drug on calcium influx. All other chemical unless mentioned were obtained from Sigma Aldrich and Invitrogen.

### Animal studies

All animal work was performed in accordance with protocols approved by the Institutional Animal Care and Use Committee of Vanderbilt University Medical Center. Immunodeficient Nu/J mice (Jackson labs) mice were used for studying the tumorigenicity. The MCF-7 (and high salt passaged MCF-7s) cells were trypsinized and washed with PBS before intramammary injection (5 × 10^5^ cells/mice) into mice. The tumor volume was calculated using the formula V = (W^(2) × L)/2 from caliper measurements, where V is tumor volume, W is tumor width and L is tumor length [34].

### Westernblot

Total proteins were extracted from cells with lysis buffer for Western blot analysis as previously described [35, 36]. Total proteins were separated on a 4–12% sodium dodecyl sulfate-polyacrylamide gradient gel and transferred onto a nitrocellulose membrane. The membranes were blocked overnight at 4° C in Tris-buffered saline with 0.05% Tween 20 (5% nonfat milk in 10 mM Tris-HCl-100 mM NaCl-0. 1% Tween 20, pH 7.4). The membranes were incubated first with Abs specific for total and phosphorylated forms at room temperature with primary Abs diluted 1 in 1,000 in blocking buffer for 2 hrs, and then with a horseradish peroxide-conjugated secondary IgG mAb diluted 1 in 5,000 for 1hr. All primary and secondary Abs were obtained from Santa Cruz Biotech (Dallas, TX). The following specific primary antibodies to Orai1 (sc-377281), STIM1 (sc-66173), P-gp (sc-55510), GADPH (sc-47724) and Actin (sc-8432) were used. The membrane was developed using the chemiluminescence kit (Millipore) and analyzed on using Bio-Rad Universal Hood II (Hercules, CA). Morphometric analysis was done using the software provided by the company.

### Quantitative real time polymerase chain reaction (qRT-PCR)

Expression profiles of genes at mRNA level in the breast cancer cell lines were analyzed using the TaqMan FAM-labeled RT-PCR primers for SIK3 (Hs00228549_m1), STIM1 (Hs00963377_m1), Orai1(Hs00385627_m1), P-gp (Hs00184500_m1), GADPH (Hs402869), and Actin (Hs4333762T), obtained from Applied Biosystems/Thermo Fisher Scientific (Grand Island, NY) as per the manufacturer's recommendation. Briefly, total RNA was extracted from 10^6^ cells using TRIzol reagent (Sigma–Aldrich) and analyzed as mentioned previously [22, 37, 38]. RNA samples were quantified by absorbance at 260 nm. The RNA was reverse-transcribed and RT-PCR (real time PCR) was performed in a final reaction volume of 20 μL using BioRad CFX96 (Hercules, CA). Each sample was analyzed in triplicate. Cycling conditions consisted of an initial denaturation of 95° C for 15min, followed by 40 cycles of 72° C for 30 s, followed by 61° C for 1 min.

### Calcium influx assay

Calcium assays were performed using Fluo-3 (Sigma Aldrich) solubilized to 10 mg/ml with dimethyl sulfoxide. Cells were loaded with 4 μg/ml Fluo-3 for 30 minutes at 37° C. Cells were washed three times in HEPES buffered saline solution (HBSS with 1 mM CaCl_2_, 0.5 mM MgCl_2_, 0.1% BSA, 10 mM HEPES) and resuspended to 1 × 10^6^ cells/ml in HEPES buffered saline solution. For measurement of store-operated Ca^2+^ influx, 3 mM EGTA and 2 mM thapsigargin were added to deplete internal calcium stores. Ca^2+^ influx was induced by subsequent addition of 2 mM Ca^2+^ (free) after store depletion [24, 39]. The emission wavelength set at (λ_em_) 485 nm and capture excitation wavelength set (λ_ex_) at 520 nm. The relative fluorescence detection was performed using FilterMax F5 spectrophotometer (Molecular Devices, Sunnyvale, CA) and data obtained using instrument software.

### Rhodamine 123 assay

P-gp activity was determined by measuring intracellular accumulation of rhodamine 123 [40]. The breast cancer cells under various treatment conditions. Briefly, cells were incubated at 37° C with 5.25 μM rhodamine 123 for 60 min. After washing in HBSS, cells were lysed in distilled water, and intracellular levels of rhodamine 123 were quantified by FilterMax F5 spectrophotometer (Molecular Devices, Sunnyvale, CA) and data obtained using instrument software. The emission wavelength set at (λ_em_) 485 nm and capture excitation wavelength set (λ_ex_) at 535 nm. Data were expressed as percentage (%) of rhodamine 123 accumulation in control cells arbitrarily set at 100%.

### Statistical Analysis

Data are expressed as mean ± SEM from five independent studies. Statistical differences between means were analyzed using a paired or unpaired Student's *t* test. A value of *p* less than 0.05 was considered significant. All data analysis was obtained using Origin 6 software (Origin Labs, Northampton, MA) or SPSS software, version 21 (IBM corporation, Armonk, NY).
